# Kalyani cohort – the first platform in Eastern India for longitudinal studies on health and disease parameters in peri-urban setting

**DOI:** 10.1017/gheg.2016.19

**Published:** 2017-02-14

**Authors:** S. Chatterjee, P. P. Majumder

**Affiliations:** 1 Biomedical Genomics Centre, PG Polyclinic Building (3rd Floor), 5 Suburban Hospital Row, Kolkata 700020, India; 2 National Institute of Biomedical Genomics, Netaji Subhas Sanatorium (2nd Floor), Kalyani 741251, India

**Keywords:** Epidemiology, genomics, health status, non-communicable disease, prospective studies

## Abstract

The Kalyani cohort created in 2010 by the National Institute of Biomedical Genomics, West Bengal, India, is designed to serve as a platform for conducting prospective basic and translational studies on epidemiology and genomics of health and disease-related parameters, particularly of non-communicable diseases (NCDs). The overall goal is to assess behavioural, biological, genetic, social and environmental factors and obtain necessary evidence for effective health improvement. Collected baseline data comprise 15727 individuals, >14 years of age from seven municipal wards in the Kalyani and Gayeshpur regions. Data are being collected on demographics, current health status, medical history and health-related behaviours. Blood samples were also collected from a subset of individuals (*n* = 5132) and analysed for estimation of known markers of NCDs. DNA has been extracted from blood samples and stored for future use. Important baseline findings include a high prevalence of diabetes, dyslipidemias and hypothyroidism. Prevalence estimates for these disorders obtained from self-reported data are significantly lower, indicating that participants are unaware of their health problems. The identification of ‘at risk’ individuals will allow formation of sub-cohorts for further investigations of epidemiological and genetic risk factors for NCDs. Access to the resource, including data and blood samples, created by this study will be provided to other researchers.

## Why was the cohort setup?

Chronic non-communicable diseases (NCDs) are the leading causes of death globally. Of the total 57 million deaths that occurred in the world during 2008, 36 million (63%) were due to NCDs, principally cardiovascular diseases (CVDs), diabetes, cancer and chronic respiratory diseases [1], with the major percentage (80%) of NCD deaths (29 million) occurring in low- and middle-income countries, mostly among individuals before the age of 60 years [2]. At the current stage of India's health transition, NCDs contribute here to an estimated 53% of deaths and 44% of disability adjusted life-years lost [3]. High prevalences of CVDs and diabetes have been recorded in India [4]; future projections are adverse [5, 6]. Though infection and malnutrition-related illnesses continue to be a major problem in many parts of India, the additional burden of NCDs is likely to strain an overstretched and resource-constrained health infrastructure [7].

While most NCDs are associated with modifiable behavioural risk factors [2], biological, social and economic factors also play an important role in determining NCD susceptibility [8]. India has a diverse population and much of the information on population health in India comes from cross-sectional medical surveys and hospital data collections. In rising to the challenge of understanding the complex causes of NCDs, longitudinal studies are a valuable tool for providing the best observational evidence of causal factors for these diseases. Such studies will also enable the establishment of repositories of biological materials (biobanks) for discovery and characterization of genes associated with common NCDs.

The Kalyani cohort study (KCS) was initiated in 2010 by the National Institute of Biomedical Genomics (NIBMG). The cohort, one of the first of its kind in the State of West Bengal, was designed to recruit 20,000 participants to create a platform for long-term prospective basic and translational studies of epidemiology and genomics of health and disease-related parameters, in particular of NCDs. The overall goal was to assess behavioural, biological, genetic, social and environmental factors and obtain necessary evidence for effective health improvement. The introduction of genetic studies in the cohort was driven by the lack of genetic epidemiological data from eastern India. Genetic analysis will have the potential to discover novel disease susceptibility loci and variants, assess association signals, and refine association signals at new and existing disease loci [9].

## Where is the cohort located and what are the population characteristics?

**Fig. 1.**
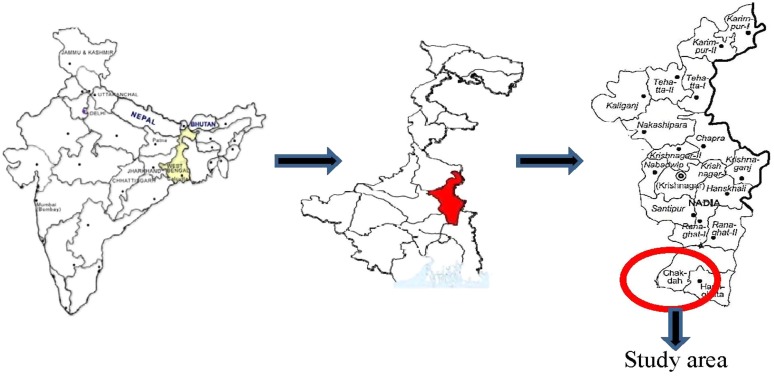
Geographical location of the Kalyani cohort.

The KCS comprises residents of the Kalyani and Gayeshpur peri-urban municipalities in Nadia district in West Bengal, a state in eastern India (Fig. 1). Nadia is situated between 22°53″ and 24°11″ North latitude and 88°09″ and 88°48″ East longitude and is about 390027 km^2^ in area. The district has eight municipalities. Kalyani and Gayeshpur lie about 50 km from Kolkata (formerly Calcutta), the capital city of West Bengal. Kalyani is considered the education and medical hub of the district and covers an area of 29.14 km^2^. According to the 2011 population census, Kalyani has a population of 100,620. Men constitute 50.6% of the population. The overall literacy prevalence of 88.8% in Kalyani is higher than the national average of 59.5%: male and female literacy prevalences are 92.8% and 84.7%, respectively. In Kalyani, 7.6% of the population is under 6 years of age compared with the national average of 11.5%. Based on the 2001 Census, the average household size of Kalyani is approximately 4.61, which is similar to the national figure (4.8) [10]. Gayeshpur municipality covers an area of 30 km^2^ and has a population of 55,048; men constitute 51% of the population. The overall literacy prevalence in Gayeshpur is 81%, with 85% of men and 76% of women being literate. Under-6's constitute 8% of the total population (http://censusindia.gov.in/2011-provresults/paper2/data_files/India2/Table_2_PR_Cities_1Lakh_and_Above.pdf). Although India contains diverse ethnic groups, the Kalyani–Gayeshpur region is by comparison ethnically homogeneous. According to the 2011 Census, 35.4% of the population in this region belongs to the scheduled castes and 2.7% to the scheduled tribes. The majority of participants in the Kalyani cohort speak the local dialect of Bengali; common tribal communities include Mundas and Oraons. This is a stable population, with virtually no immigration and little emigration, hence insuring little loss to follow up.

## What does the cohort cover?

The KCS aims to provide longitudinal, self-reported and measured data to assess the prevalence and distribution of chronic NCDs and their risk factors in the study population. The cohort, that currently comprises only adults, is being expanded to include children. This will enable trans-generational assessment of predispositions to NCDs and of childhood vulnerabilities. Another distinctive feature of this cohort is the collection of blood samples for biochemical evaluation and development of a large population-based bio-bank. This will therefore provide a resourceful research platform for investigating the role of genes, environment and lifestyle on various health conditions and assisting in maximizing the effectiveness of strategies for NCD prevention, early detection and management.

## Who is in the KCS?

There are 21 wards (administrative units) in the Kalyani municipality and 18 wards in the Gayeshpur municipality, with a total population of 155,668 [10] (http://censusindia.gov.in/2011-provresults/paper2/data_files/India2/Table_2_PR_Cities_1Lakh_and_Above.pdf). A statistical sampling design was used to establish the Kalyani cohort from the residents of these two municipalities. The sampling design was based on probability proportional to census (2001) population size, so that a resident of a larger ward has a proportionately higher chance of being selected in the study ensuring better representativeness of the entire study area. According to the 2001 census [11], which was the last census data available at the time of initiation of the study, the total number of residents in Kalyani and Gayeshpur municipalities were 82,135 and 55,048 and the total number of households in the two municipalities was 18,489 and 11,567, respectively. To select the 20,000 participants, a proportional allocation of 12,123 and 7992 participants was made to Kalyani and Gayeshpur municipalities, respectively. Assuming that we would select three adults from each household – the head of the household, his spouse and an offspring – we had to select about 4700 households from Kalyani municipality and about 2300 households from Gayeshpur municipality. Based on the average number of households (about 1000) in the wards of the two municipalities, we decided to randomly select seven wards; four from Kalyani (Wards 2, 9, 17 and 18) and three from Gayeshpur (Wards 2, 5 and 8) municipalities. These seven randomly selected wards, when we conducted an actual survey, had a total of 5733 households; all households in each sampled ward were selected. Within each household, all family members aged >14 years were eligible for recruitment into the study. A non-responder household was defined as a household whose members declined to participate even after being contacted on at least three occasions. Blood samples were collected from 5132 participants using the systematic sampling design to ensure representativeness; attempt was made to collect at least two unrelated individuals from every fifth household.

The study was approved by the *Committee for Protection of Research Risks to Humans* of the NIBMG. Data and blood sample collection was carried out after obtaining written, informed and broad consent from each participant, including of follow-up. Broad consent allows the use of data and blood samples for future research with the clear understanding that these will be treated with care to protect the donors from future research risks, with the usual monitoring of usage by the institutional ethics committee. For participants between 14 and 18 years of age, written consent was taken from one of the parents, after explaining the purpose of the study to both the participant and her/his parent.

## How are they being followed up?

Each KCS participant is contacted by a field investigator and her/his family is visited. Data are collected in the homes of the participants, maintaining privacy to the extent feasible. Prior to data collection, all field-investigators (most of whom are graduates in social science) were imparted training. Field supervisors oversee the data collection. Professional phlebotomists collect blood samples using disposable needles and syringes. Between 5 and 8 ml of blood is collected from each participant.

Participants, who were recruited in the first phase of the study in December 2010, were re-surveyed in their first follow-up from December 2012. The first follow-up was completed in April 2016. A comprehensive follow-up is being targeted every 2–3 years and efforts are being made to contact participants in approximately the same order as they were interviewed to maintain the 2–3 year follow-up time. Field investigators of the KCS team visited selected households and interviewed study participants with a questionnaire. The same questionnaire that was used for collecting baseline data was also used in the first follow-up. This allowed baseline variable information to be followed over time, whilst allowing further collection of information on new exposures and interim health history, including cancer, CVD, stroke and other chronic diseases that occurred since the last contact. Recorded information was then cross-checked by the field supervisor to avoid possible errors and omission of events. Attrition due to mortality, migration, non-cooperation and migration due to new building constructions are being tracked and recorded.

**Fig. 2.**
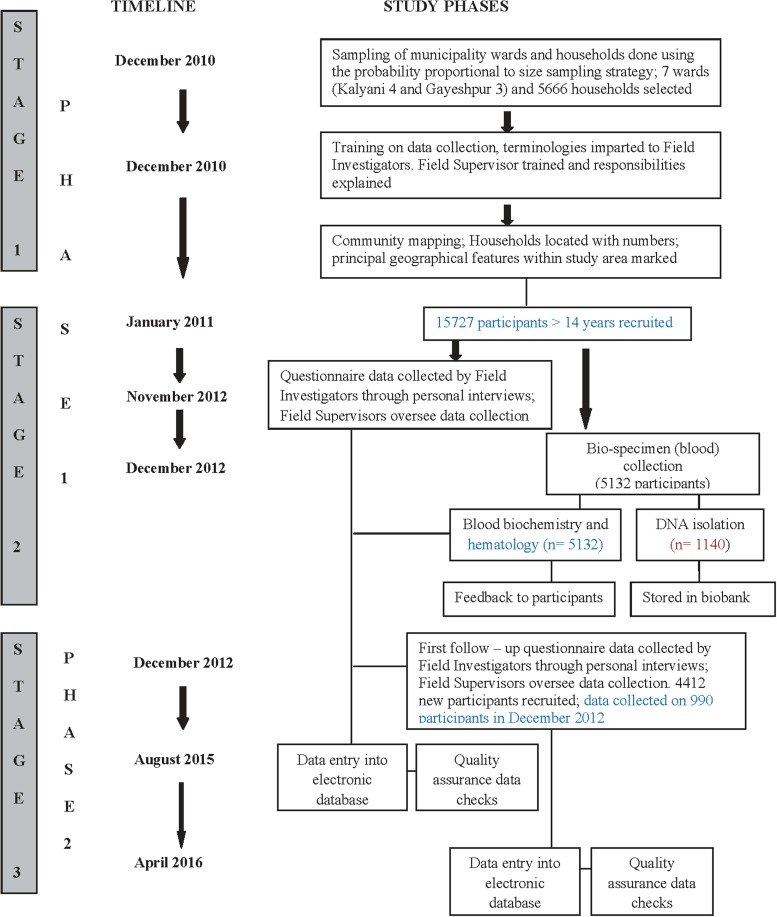
The Kalyani cohort study phases and its timelines.

To date, 353 participants have died 1196 participants have migrated, 115 participants refused to co-operate in the second phase and 213 female participants married and relocated elsewhere. For migrated participants, re-contacting efforts are being made. The field supervisor personally contacted participants who initially refused to cooperate, to understand reasons for non-co-operation and provide them with explanations if any. The vast majority of the initial refusals were successfully resolved by sustained discussion and providing of further information about the study. The study phases and timelines are demonstrated in Fig. 2.

The study sample was augmented in the second phase with 4412 new recruits. These included new participants within same selected households and also from families who may have migrated into the study area and were living in sampled households.

## Statistical power

We assessed the statistical power attainable for the sample size of 15,727 individuals. If the population prevalence of a trait/disorder is between 10% and 40%, then with this sample size it will be possible to estimate the population prevalence to within at least ±0.09% of the true prevalence with a 95% level of confidence. Further, with this sample size – and assuming a 1:9 ratio of cases to controls, an exposure prevalence of 30% among controls, and alpha = 5% – the study has >90% power of detecting an odds ratio of at least 1.2. Under the same scenario, if the exposure among controls is 20%, then the statistical power reduces to about 80%.

## What has been measured?

### Questionnaire and interview

Trained field interviewers used structured questionnaires and conducted in-depth face-to-face interviews at participants’ homes. The questionnaire, developed by the NIBMG, Kalyani, with assistance from anthropologists, statisticians and biomedical scientists, was used to collect information on demography, occupational history, self-rated mood, anxiety and depression, food habits, nature and frequency of tobacco and alcohol use, physical activity, personal medical history and current health condition. The questionnaire was first field-tested and validated by comparing information collected by two independent field investigators on the same study participant. Listing of data items collected using a structured questionnaire is provided in Table 1. Geographical mapping was done manually and hand-drawn maps were prepared that precisely located all households and demarcated ward boundaries and principal geographical features within the study area.

**Table 1.** Listing of data items collected in the Kalyani cohort study

	Variable	Variable details	Phase I 2010–2012	Phase II 2012 – ongoing
1	Socio-demographics	Year of birth	x	
		Gender	x	
		Residential address	x	x
		Marital status	x	x
		Occupation	x	x
2	Lifestyle habits	Dietary habits	x	x
		Physical activity	x	x
		Tobacco smoking	x	x
		Other forms of tobacco intake	x	x
		Alcohol consumption	x	x
3	Mental health	Anger, irritation, anxiety	x	x
		Other stressful situations	x	x
4	Medical history	Vaccination	x	x
		Chronic diseases (diabetes, hypertension, CVDs, and cancer, asthma)	x	x
		Infectious diseases (chicken pox, measles, malaria, typhoid, polio, diarrhoea, tetanus, tuberculosis, dermatological infections and ear infections)	x	x
		Others (gastrointestinal, haematological, neurological, endocrinal, dental, obstetric and gynaecological)	x	x
5	Current health and symptoms	Chest pain, chest discomfort, breathlessness, sweating, wheezing in chest	x	x
		Extreme thirst, increased urinary frequency, delayed wound healing	x	x
		Back pain, other joint pain	x	x
		Tiredness, fatigability	x	x
		Current intake of medications	x	x

### Blood collection and baseline measurements

Blood samples were collected by venipuncture from study participants, after a 12-h overnight fast. All blood samples were drawn by trained and certified phlebotomists who had undergone mandatory training and periodic skill-verification from a reputed laboratory accredited by the National Accreditation Board for Laboratories, Government of India. Blood parameters measured included fasting glucose, lipid profile, renal profile, full blood count, liver function tests and thyroid stimulating hormone. A repeat blood sample was collected on 200 randomly allocated participants and blood parameters were measured. Results on these blood samples were used to validate results of the first-phase blood collection.

DNA has been extracted from the first 1140 blood samples (95 male and 95 female samples randomly selected from each of six wards: Kalyani wards 2, 9 and 17; and Gayeshpur wards 2, 5 and 8); extractions from the remaining blood samples are in progress.

### Blood sample storage

Samples of EDTA–blood were transported from field sites under optimum conditions and were banked in the NIBMG laboratory in −20 °C freezers, using standardized blood sample storage protocols and following best practices for specimen storage and retrieval. All stored blood samples have a unique identifier and are labelled with a printed label. The freezers are operated using effective facility environments that include ambient temperature controls, good air circulation, lighting and security. Emergency preparedness plans are in place to cover equipment failures and power interruption that include back-up power supplies such as diesel generators. Written Standard Operating Procedures are in place to respond to freezer failures and other emergency situations.

The blood samples are maintained in a stabilized state as much as possible, to avoid frequent freeze/thaw cycles. Stored blood samples are thawed and DNA extracted from stored blood samples are divided into multiple aliquots and stored in cryovials (at −80 °C). Blood serum samples are however not being stored in the current phase.

### Data entry and management

All data were collected on printed questionnaires. Each questionnaire was manually scrutinized for missing and incoherent data. Scrutinized data were entered in a computer database, after deleting personal identifiers. Data-entry was carried out under supervision. Statistical cross-checking of various fields of data in the database are periodically carried out. These checks include missing variables, out-of-range, etc. Inconsistencies are resolved by re-contacting the participants.

### Quality assurance

Tiered monitoring of primary data collection was done on a random-sampling basis.

About 30% of primary data were monitored during collection and cross-checked by the field supervisor. Inconsistencies were resolved in the field. About 10% of the primary data were cross-checked by the field co-ordinator, through household visits. About 5% of the participants were re-contacted by the study manager for discussion of clinical symptoms and primary data were cross-checked during these discussions. The study manager also monitored data inconsistencies between the two rounds of data collection. Most inconsistencies were resolved by re-contacting participants.

Blood samples collected on 4932 participants in the first phase were tested in a reputed pathological laboratory. The pathological and biochemical results on the first set were analysed. A second phase of blood sample collection was done on 200 randomly drawn participants from whom blood was not drawn in the first phase. These 200 blood samples were tested in a different pathological laboratory of repute. Results of both set of samples were compared for consistency. Two blood parameters (ALT and AST) were found to lack consistency beyond acceptable threshold; these two parameters are, therefore, being re-assayed before inclusion in the database. Efforts are being made to improve data quality on this parameter.

*Feedback of baseline results to participants*: The original results from the baseline blood tests were handed over to each participant after scanning and uploading the reports into an electronic database. The reports were personally distributed to the participants by the field investigators, field supervisor, study coordinator and the study manager. Results were accompanied by a basic explanation of any abnormal findings, lifestyle and health guidance and advice to consult physicians for those with abnormal reports for further investigations and treatment.

## What has been found?

**Fig. 3.**
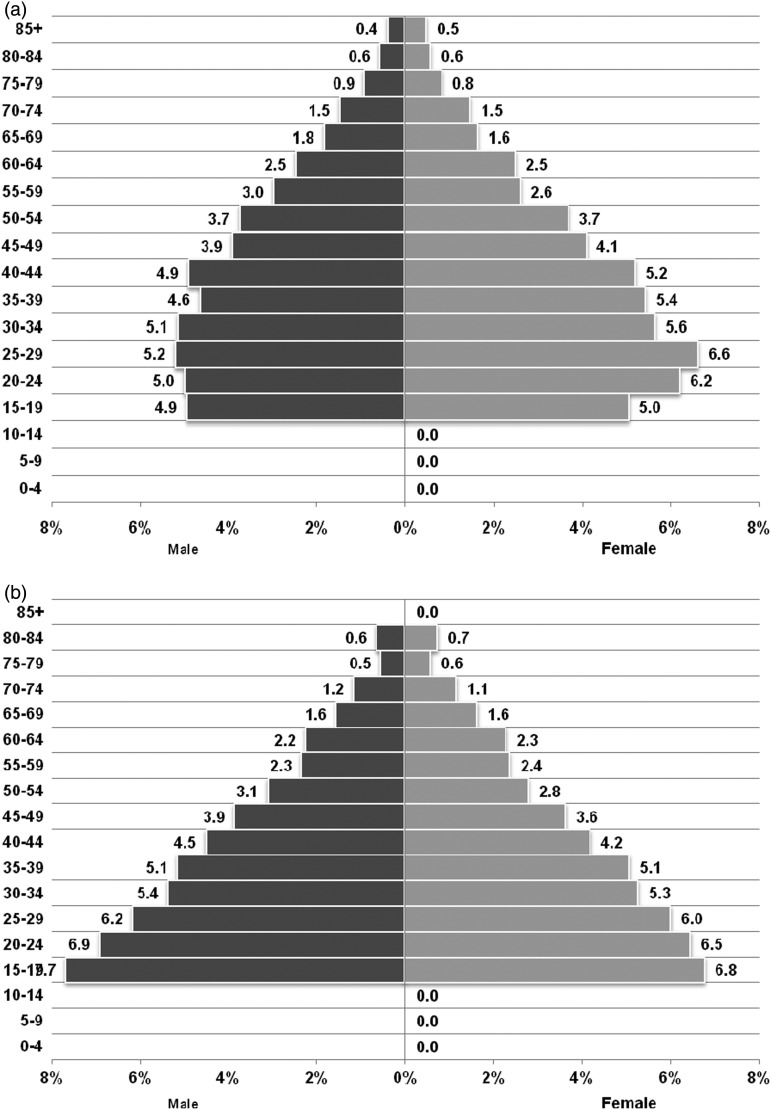
(*a*) Population pyramid of the Kalyani cohort. (*b*) Population pyramid of 2011 population census data.

A high level of participation was observed in the KCS. Out of 5733 households who were invited to participate in the baseline study, 67 refused to participate resulting in a response of 98.8%. Whenever a non-responder was encountered, the entire household was withdrawn from the study. The cohort now has a total of 15,727 participants aged >14 years (Kalyani *n* = 9031; Gayeshpur *n* = 6696; 7549 men and 8178 women) from these households in the first phase. Selected characteristics of the participants in the cohort as obtained from our baseline survey are listed in Table 2. The overall mean age at baseline was 40 ± 17.1 years (men 40.7 ± 17.2 years; women 39.4 ± 16.9 years). The majority of the Kalyani cohort members were married. The age–sex pyramids (Figs.3*a* and 3*b*) indicate a gradually ageing population with more than 10% of the population aged 65 years and above and how it compares with the 2011 population census data. Around 10% of the population belonged to the 40–44-year age group, with the greatest proportion of women in the age group 20–49 years. Most men were on salaried jobs working in governmental and non-governmental offices, while the majority of women were housewives. The majority (92.3%) of our participants reported having their major meals at home; however, just under half (47.7%) reported extra salt consumption in their diet regularly; fast food intake (snacks) ≥2 times/week was noted among 61.7% of participants. Nearly three-quarters (73%) of the study population never participated in any physical activity. One-fifth of the sampled population were current smokers. Alcohol consumption was noted only among 10% of participants, which is likely to be due to underreporting because of the social stigma associated with alcohol consumption.

**Table 2.** Characteristics of participants of the Kalyani cohort at baseline (2010–2012)

Variable	*n*	%
Sex		
Male	7549	48
Female	8178	52
Age groups		
<20 years	1570	10
20–40 years	7219	45.9
41–60 years	4727	30.1
61–80 years	1926	12.3
>80 years	285	1.8
Area of residence		
Kalyani municipality	9031	57.4
Gayeshpur municipality	6696	42.6
Marital status		
Married	10451	66.5
Unmarried	4027	25.6
Widowed	1188	7.6
Divorced	50	0.3
Separated	10	0.1
Occupation		
Farmer	176	1.1
White-collar work	2188	13.9
Business	1536	9.8
Professional	84	0.5
Student	2250	14.3
Housewife	5515	35.1
Others (daily waged labour, unemployed, retired)	3969	25.2
Tobacco smoking		
Former smokers/never smokers	12566	79.9
Current smokers	3160	20.1
Other forms of tobacco users		
Former tobacco users/never tobacco users	13014	82.8
Current tobacco users	2712	17.2
Alcohol consumers		
Former consumers/abstainers	14127	89.8
Current consumers	1592	10.2
Physical activity		
No participation	11501	73.1
Irregular participation	632	4.02
Participation 1–2 h/week	61	0.4
Participation >2 h/week	3527	22.4
Diet		
Household food usually (Yes)	14519	92.3
Fast food/snacks consumers (Yes)	9701	61.7
Intake of extra salt with food (Yes)	7496	47.7

At baseline, 62.6% of the population did not report any chronic disease. The most commonly reported chronic condition was CVDs, prevalent in 19% of the participants. The second most commonly reported condition was arthritis and joint pains (14.4%), followed by diabetes (6.5%). Significantly higher proportion of women, compared with men, reported CVD, diabetes and joint pains. Table 3 presents baseline prevalence of self-reported chronic conditions and the *p* values for test of equality of proportions between men and women.

**Table 3.** Baseline prevalence of self-reported chronic conditions in the Kalyani cohort

Chronic conditions	*n* (%)	Male (%)	Female (%)	Gender difference *p* value
CVD (includes heart failure, hypertension and coronary heart disease)	2995 (19)	1263 (42.2)	1732 (57.8)	<0.0001
Diabetes	1016 (6.5)	527 (51.9)	489 (48.1)	0.01
Dyslipidaemias	99 (0.6)	47 (47.5)	52 (52.5)	0.9
Asthma	652 (4.2)	322 (49.4)	330 (50.6)	0.5
Cancer	418 (2.7)	114 (27.3)	304 (72.7)	<0.0001
Neurological diseases (includes CVAs (cerebrovascular accidents))	928 (5.9)	436 (46.9)	492 (53.0)	0.5
Arthritis and joint pains	2260 (14.4)	726 (32.1)	1534 (67.9)	<0.0001
No chronic diseases	9850 (62.6)	5045 (51.2)	4805 (48.8)	<0.0001

Anaemia (defined as Hb < 13gm/dl among men and 12 gm/dl among women) was noted in 28% of men (mean Hb (±s.d.) − 13.9(±1.6) gm%) and 45.9% of women (mean Hb (±s.d.) – 12.2(±1.2) gm%). Studies have shown a higher prevalence of anaemia in India affecting all sections of the society [12]. According to the 2005–2006 National Family Health Survey (NFHS-3), a household survey aimed at having national and state representative data on population health and nutrition, the prevalence of anaemia was 55% among women aged 15–49 years and 24% among men aged 15–49 years [13].

**Fig. 4.**
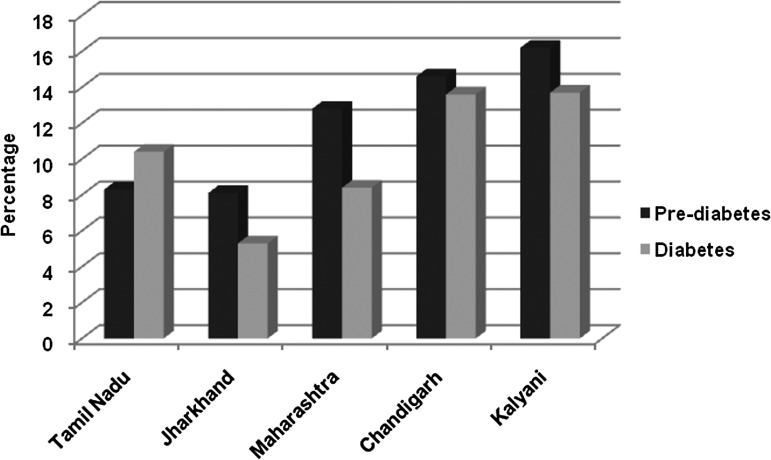
Prevalence of diabetes and pre-diabetes in the Kalyani cohort compared with other Indian regions.

Baseline measurement of fasting glucose level revealed 13.9% of participants had diabetes (fasting blood glucose ≥ 126 mg/dl) and 16.2% had pre-diabetes (fasting blood glucose ≥ 110 mg/dl and <126 mg/dl) [14]; these proportions did not differ significantly between males and females in our population (*p* = 0.06). The estimates were high compared with prevalences reported from other parts of India (Fig. 4) in the INDIAB study that provides weighted prevalences of diabetes [15]. Self-reported diabetes in our cohort was 6.5%. Disparate findings noted between self-reported diabetes and diabetes prevalence according to laboratory estimates (6.5% *v.* 13.9%) is of concern because individuals who are unaware of their disease status are left untreated and are thus more prone to microvascular as well as macrovascular complications of diabetes. The higher prevalence of pre-diabetes is also a cause of worry, as this implies a population at risk of developing diabetes in the near future.

Hypercholesterolaemia (total cholesterol ≥ 200 mg/dl) [16] was noted among 24.4% of the participants; hypertriglyceridaemia (triglyceride ≥ 150 mg/dl) among 37.8%, low high-density lipoprotein (HDL)-cholesterol (HDL-C levels <40 mg/dl for men and <50 mg/dl for women) among 16% of men and 98% of women, and high low-density lipoprotein (LDL) (LDL ≥ 130 mg/dl) among 20.2% of the population. A significantly higher proportion of women had adverse cholesterol, triglycerides, LDL and HDL levels in our sample, compared with men (*p* = 0.04). At least one lipid abnormality was present in 76% of the participants. Although regional disparity in prevalence of dyslipidaemia has been noted in India [17], its prevalence in the Kalyani cohort is alarming. Dyslipidaemia (defined by at least one abnormal lipid profile) has been closely linked to the pathophysiology of CVD and is a key independent modifiable risk factor for CVDs.

Hypothyroidism (TSH ≥ 5.5 µIU/l) prevalence among men (11.7%) and women (15.4%) was high compared with national estimates. According to the 2000–2001 survey conducted by the National Nutrition Monitoring Board (NNMB) in rural areas of Kerala, Tamil Nadu, Karnataka, Andhra Pradesh, Maharashtra, Madhya Pradesh, Orissa and West Bengal, the overall prevalence of goitre (iodine deficiency disorder) was highest in Maharashtra (11.9%) and West Bengal (9%) [18]. The high prevalence of TSH in our sampled population is also of concern as iodine deficiency could manifest in several other ways such as cretinism, impaired cognition, and impact early brain development and learning abilities in children. Biochemical findings of the cohort participants are summarized in Table 4.

**Table 4.** Abnormal laboratory parameters in Kalyani cohort overall and according to gender

	Overall		Male		Female	
Blood parameters	*n*	%	*n*	%	*n*	%
Diabetes (fasting blood glucose ≥ 126 mg/dl)^a^	707	13.9	335	14.8	372	13
Pre-diabetes (fasting blood glucose ≥ 110 mg/dl and <126 mg/dl)^a^	835	16.15	337	14.9	498	17.4
Dyslipidaemia (presence of at least one lipid abnormality)	4069	76.75	1229	54.3	2840	99.2
Hypercholesterolaemia (total cholesterol ≥ 200 mg/dl)^b^	1227	24.4	482	22.8	745	26
Hypertriglyceridaemia (triglyceride ≥ 150 mg/dl)^b^	1909	37.75	951	42.1	958	33.4
Low HDL (HDLC < 40 mg/dl for men and <50 mg/dl for women)^b^	3198	57	362	16	2836	98
High LDL (LDL ≥ 130 mg/dl)^b^	959	20.15	258	15.8	701	24.5
Hypothyroidism (TSH ≥ 5.5 µIU/l)^c^	706	13.55	264	11.7	442	15.4

a World Health Organization Report [15].

b NCEP Report [17].

c Unnikrishnan *et al*. [19].

## What are the key strengths and weaknesses?

One of the main strengths of this study is the establishment of a large population-based cohort with comprehensive information on demographics, lifestyle factors, biochemical and biological parameters, thus providing a rich database and an attractive platform for health researchers and public health practitioners to conduct cross-sectional epidemiological studies on various health-related topics. The longitudinal nature of the study will enable periodic up-dating of information on new exposures and health-related conditions, and repeat measures on selected variables of research interest. The study did not have an upper age limit for participants and therefore will be able to investigate risk factors for diseases in the older populations. The statistical sampling method of the population will help broaden the generalizability of findings beyond the local study population to the wider peri-urban population in India. Collection and bio-banking of blood samples from study participants will provide a valuable resource for molecular epidemiological studies for NCD risk assessment and early detection. Notably, the KCS is one of few cohort studies in India to have collected and stored DNA samples for studying genetic susceptibility to chronic disease. The KCS team has fostered a close relationship with the community and study participants which we believe will help us ensure high retention and good response rates to sensitive questions.

A major weakness of the study is the lack of anthropometric measurements on the participants of our cohort. Several studies have reported strong and independent associations between body mass index and NCDs; by contrast, our present data will not permit us from studying such associations. We do plan to collect data on these variables in the follow-up surveys. A further limitation of our study is that most data are self-reported, which require a more stringent level of rigour for validation in order to reduce potential biases or mis-reporting. We plan to introduce this in the near future. However, relying on self-reported data is common in observational studies, and research has shown that self-report of major medical conditions correlates well with medical records [19, 20]. We plan to overcome this limitation in future by collecting prospective outcome data. Deaths that were recorded during follow-up visits were not subjected to verbal autopsy; this prevented a systematic investigation of the causes of death in our population. We plan to introduce WHO verbal autopsy from the next round of data collection. Although a system of registering births and deaths exists in both Kalyani and Gayeshpur municipalities, linking of the cohort data with these government registration data was not possible because of the large time lag between collection of vital statistics by the government and their availability as electronic data. In this study, although 100% manual checking of entered data was done, double-entry of data or electronic data capture was not done; this is a further limitation. We plan to introduce an electronic data capture system soon.

Though DNA isolation has been done, blood serum was not banked; thus the possibility of evaluating diagnostic or prognostic markers in the serum is compromised. Our laboratory analysis of blood also did not include HIV testing. Due to limitations of funds, we also could not collect other types of biological samples (e.g. urine, saliva and stool samples). We expect to overcome all these limitations in the future.

## Future plans

We plan to expand the KCS to include babies and children of the families already recruited into the study. For any participant below 18 years of age, a signed informed consent will be taken from the parents consenting to their child's participation in the research. Additionally, for all participants between 12 and 18 years of age, an assent for participation will also be taken. The main purpose for lowering the recruitment age is to obtain estimate of preterm birth, understand the health of the children, in relation to the health of their mothers, vaccinations taken, and other variables that are relevant to child health. We hope that the proposed expansion of the KCS will contribute to a better understanding of health and disease and to betterment of health planning of this region, through (a) identification of transmitted factors, both genomic and environmental, and (b) prevention of disease in children by genetic testing and diagnosis.

Our future plans also include replacing paper questionnaires with tablets for direct electronic data collection. We shall include anthropometric measurements, blood pressure and such other variables that are informative of the health status of an individual and can be non-invasively measured. We plan to form sub-cohorts for in-depth studies on specific phenotypes. For example, we will follow up women who become pregnant and follow them up through the period of their pregnancies and collect multi-dimensional data – observational, biochemical and genetic. The outcome of each of these pregnancies, such as preterm birth, birth-weight and length of the child, etc. will be correlated with the multi-dimensional data to assess risk factors that are associated with specific features of a birth outcome. We also plan to study responses to vaccination, especially those that are mandated by the Government of India. These responses will be studied primarily, but not exclusively, by estimating relevant immunological correlates of vaccine response. In the first round of collection of blood samples, only DNA was extracted from each sample. In the future rounds of blood collection, we shall collect the each blood sample in such a way that serum and plasma can also be collected, aliquoted and stored. We will also attempt to collect other bio-samples, such as saliva, stool, urine, nail, etc. Additional infrastructure, especially freezer space, required for this purpose is being created. Finally, we will attempt to augment the cohort to offset non-response, withdrawals and losses to follow up. Non-responders were all households; not individuals within households. Usually, when we were able to gain the trust of some members of a household, all members of the household co-operated; otherwise, no members of the household were indicated willingness to participate. So far, 67 (1.2%) of the households selected declined to participate. We will soon add 67 households from an adjacent ward.

## Governance and resource access

A Cohort Advisory and Monitoring Committee (CAMC) shall be set up to monitor progress of the project and to provide advice on mitigation of challenges, expansion and review of new proposals that will use the resource created by the cohort. This Committee will include members with expertise in medicine, public health, genetics, management, computer science and research ethics. The resource created by this cohort study, including data and blood samples, will be open for access by other scientists, with priority accorded to those who are directly involved with this study. For accessing and using the resource for non-commercial scientific activities, a written proposal clearly indicating how the resource will be used should be submitted to the Secretary, CAMC at secy.camc_kcs@nibmg.ac.in by the intending user, who shall be a scientist in good standing. This proposal will be evaluated by the CAMC and, if approved, access to the resource will be provided.

## Conclusion

The KCS is a large peri-urban population cohort in India. This resource will serve as a platform for genetic epidemiological and natural history studies on various traits and diseases. This study has brought into focus the high prevalence of some major risk factors for chronic NCDs within the study population. The study data will allow further investigations of the epidemiological and aetiology of NCDs, including genetic susceptibility. Access to the KCS resource, including data and bio-samples, will be provided to other researchers. A plan for future expansion of the cohort and creation of defined sub-cohorts is proposed.
